# Method for high frequency tracking and sub-nm sample stabilization in single molecule fluorescence microscopy

**DOI:** 10.1038/s41598-018-32012-1

**Published:** 2018-09-17

**Authors:** Patrick D. Schmidt, Benjamin H. Reichert, John G. Lajoie, Sanjeevi Sivasankar

**Affiliations:** 1 Department of Electrical and Computer Engineering, Iowa State University, Ames, IA 50011 USA; 2 Department of Physics and Astronomy, Iowa State University, Ames, IA 50011 USA; 3 Present Address: Department of Biomedical Engineering, University of California, Davis, CA 95616 USA

## Abstract

While fluorescence microscopes and atomic force microscopes are widely used to visualize, track, and manipulate single biomolecules, the resolution of these methods is limited by sample drift. To minimize drift, active feedback methods have recently been used to stabilize single molecule microscopes on the sub-nanometer scale. However, these methods require high intensity lasers which limits their application in single molecule fluorescence measurements. Furthermore, these feedback methods do not track user-defined regions of the sample, but rather monitor the relative displacement of an unknown point on a fiducial marker, which limits their use in biological force measurements. To overcome these limitations, we have developed a novel method to image, track and stabilize a sample using low laser intensities. We demonstrate the capabilities of our approach by tracking a user-chosen point on a fiducial marker at 8.6 kHz and stabilizing it with sub-nanometer resolution. We further showcase the application of our method in single molecule fluorescence microscopy by imaging and stabilizing individual fluorescently-tagged streptavidin proteins under biologically relevant conditions. We anticipate that our method can be easily used to improve the resolution of a wide range of single molecule fluorescence microscopy and integrated force-fluorescence applications.

## Introduction

Imaging, tracking and manipulation of single biological molecules are widely used to obtain key mechanistic insights into biological processes. Single molecule fluorescence microscopes are routinely employed to localize and track proteins with nanometer spatial and millisecond temporal resolution^1–5^. Interactions between single biological molecules are also commonly measured using mechanical probes such as atomic force microscopes (AFMs)^6–9^. In fact there have been several recent attempts at combining single molecule AFM and fluorescence microscopy in order to measure force-induced changes in the conformation and activity of single proteins^10,11^ and to ‘cut and paste’ single fluorescent biomolecules for the bottom-up assembly of nanoscale structures^12–15^.

Despite their widespread use, the resolution of single molecule AFM and fluorescence methods is severely limited by thermal and mechanical drifts of the microscope and sample. This drift is particularly pronounced in fluid environments and at ambient temperatures: conditions which are essential for biologically relevant measurements. For instance, while it is theoretically possible to localize a single stationary fluorescent molecule with sub-nanometer accuracy, drift of both the microscope stage and the molecule itself blurs the fluorescence image and reduces localization precision. Similarly, when probing a single biomolecule with AFM, undesired drift of the sample and the AFM tip makes it impossible to repeatedly address the same molecule. Drift has also made it particularly difficult to develop instrumentation that integrates single molecule AFM and fluorescence-based measurements in a high-throughput fashion.

Since cryogenic temperature measurements, which have traditionally been used to combat undesired drift^16,17^, are not compatible with biological experiments, several active stabilization methods for microscopes have recently been developed^18–23^. Of these techniques, back-scatter based active stabilization provides the high spatial and temporal resolution required to track molecular dynamics. Back-scatter based active stabilization platforms, which are commonly called ultrastable microscopes, typically reflect a laser off a sample fiducial and detect the backscattered light using a position sensitive detector. To map detector signals to drift, the fiducial is initially scanned through a known area and the detector response is correlated to fiducial displacement via a polynomial fit for each spatial dimension^18^. With the detector signal correlated to fiducial displacement, sample movement is monitored in real-time and the sample is stabilized by moving the microscope stage. While back-scatter based active stabilization achieves impressive angstrom-level stability over a significant bandwidth (0.1–10 Hz), it requires high laser intensities which quickly and irreversibly bleach fluorescent dyes. This precludes the use of this active-feedback method in single molecule fluorescence microscopy, which requires significantly lower laser intensities. Furthermore, while the mapping technique used in back-scatter based active stabilization provides high displacement resolution, it does not lend itself to pinpointing a specific feature on the fiducial. For instance, when this method has been used to stabilize an AFM^18^, the AFM tip is not directly imaged, and as such the precise location of the tip apex is unknown. Consequently, the location of the AFM tip apex is determined by scanning the tip over a hard feature on the sample. This limits the use of this feedback method in AFM based single molecule force measurements, where the tip is functionalized with soft biological molecules that can be easily destroyed by compressive forces during scanning.

To extend active feedback to single molecule fluorescence applications, we have developed an optical feedback method that uses laser intensities which are several orders of magnitude lower than existing ultrastable methods. Instead of using a fitting approach to map fiducial drift, we utilize a simple discrete map to track fiducial motion with high temporal resolution. Since we directly image the fiducial, our method allows us to stabilize a user-selected point, thereby adding high accuracy to the precision of ultrastable microscopy. We demonstrate the capabilities of our method by stabilizing a user-defined point on a fiducial with sub-nanometer resolution and at a tracking rate of 8.6 kHz. To showcase its application in single molecule fluorescence microscopy, we demonstrate this method by stabilizing individual fluorescently-tagged streptavidin proteins under biologically relevant conditions.

## Methods

### Imaging a user-defined point on a fiducial

**Figure 1:**
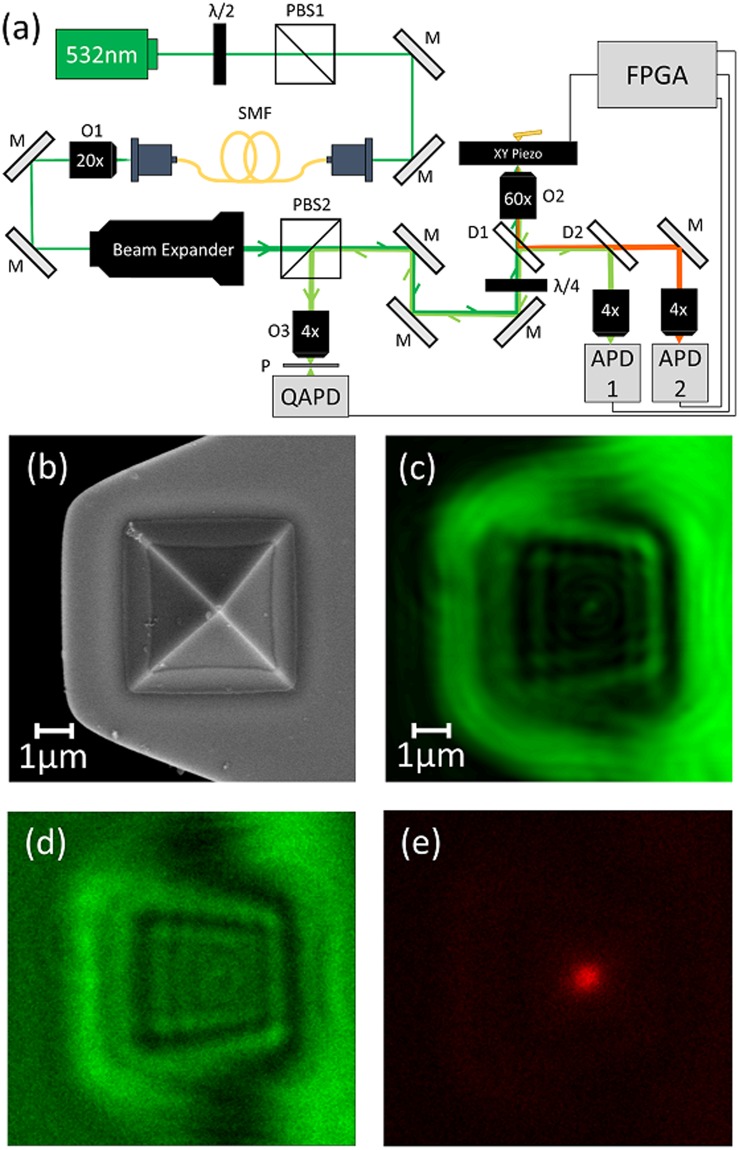
Experimental setup and demonstration. (**a**) Setup schematic. λ/2- half waveplate, PBS- polarizing beam splitter, M- mirror, SMF- single mode fiber, O- objective, λ/4- quarter waveplate, P- pinhole, QAPD –quadrant avalanche photodiode, D- dichroic. (**b**) SEM image of an AFM tip identical to the one used for feedback. Backscattered image of an AFM tip (10 µm × 10 µm) simultaneously acquired by **(c)** QAPD and **(d)** APD 1. **(e)** Tip luminescence simultaneously imaged by APD 2. The excellent agreement between the backscattered and fluorescence images demonstrate that the QAPD sum channel produces a faithful image of the AFM tip apex.

Since previous ultrastable techniques have focused on active stabilization of an AFM tip^18,19^, we directly benchmarked our method using an AFM tip as a fiducial for sample stabilization. An AFM cantilever (Oxford Instruments, TR400PSA) was glued to a coverslip and mounted on a custom confocal fluorescence microscope^24^, with the apex of the AFM tip pointed towards the objective (Fig. 1(a)). For reference, an SEM image of an identical tip is shown in Fig. 1(b). A 532 nm laser, coupled to a single mode fiber, was circularly polarized and focused on the AFM tip with a high numerical aperture (1.42) oil immersion objective. The low intensity laser light (4-5 µW) used in our experiments is compatible with single molecule fluorescence microscopy. The piezoelectric sample stage (Physik Instrumente, P-733.3, E-712 controller) raster-scanned the fiducial through the focused laser, and light backscattered off the tip was collected by the objective and directed through a quarter waveplate and polarizing cube beam splitter, splitting the backscattered and incident light^18^. Backscattered light was then focused by a 4X objective through a 50 μm pinhole onto the detection face of a quadrant avalanche photodiode (QAPD) (First Sensor, QA4000-10) operated in proportional mode. Using a QAPD rather than a conventional quadrant photodiode (QPD) allows for detection of reflected laser light that is several orders of magnitude lower in intensity, making this detection scheme ideal for low light fluorescence applications.

The QAPD was mounted on a custom printed circuit board which converted the APD quadrant currents into voltages, and output three voltage values: total current from the sensor, the difference between the left and right halves of the sensor, and the difference between the bottom and top halves of the sensor. A field programmable gate array (FPGA, National Instruments, USB 7856-R utilizing a Xilinx Kintex-7 K160T chip) rapidly digitized each value with an onboard multichannel successive approximation register ADC and produced two difference signals. The difference signals were normalized by dividing each by the total (sum) signal to eliminate sensitivity to intensity fluctuations. These normalized values yield the 2D position of the reflected laser on the face of the detector, where the laser position on the detector face varies deterministically with the 2D position of the sample fiducial.

Transmitted backscattered light was reflected by a second dichroic and focused by a 4X objective on the detection face of a single photon-counting avalanche photodiode (APD, Micro Photon Devices). The photon counts were timestamped with the FPGA’s onboard clock and stored in onboard registers. This allowed direct imaging of the fiducial produced by the sum channel of the QAPD and the APD (Fig. 1(c) and (d)). Furthermore, because the AFM tip is made of silicon nitride, which is known to luminesce at a longer wavelength when excited with 532 nm light^25,26^, an image of the AFM tip was simultaneously generated by using a 4X objective to focus the luminescence on a second APD (Fig. 1(e)). As described in the results section, simultaneously acquired back-scattered (Fig. 1(c) and (d)) and luminescence images of the AFM tip (Fig. 1(e)) permitted us to unambiguously identify the tip apex. In subsequent measurements, we used the tip apex as a fiducial, generating high resolution maps for tracking the tip.

### Tracking fiducial position

**Figure 2:**
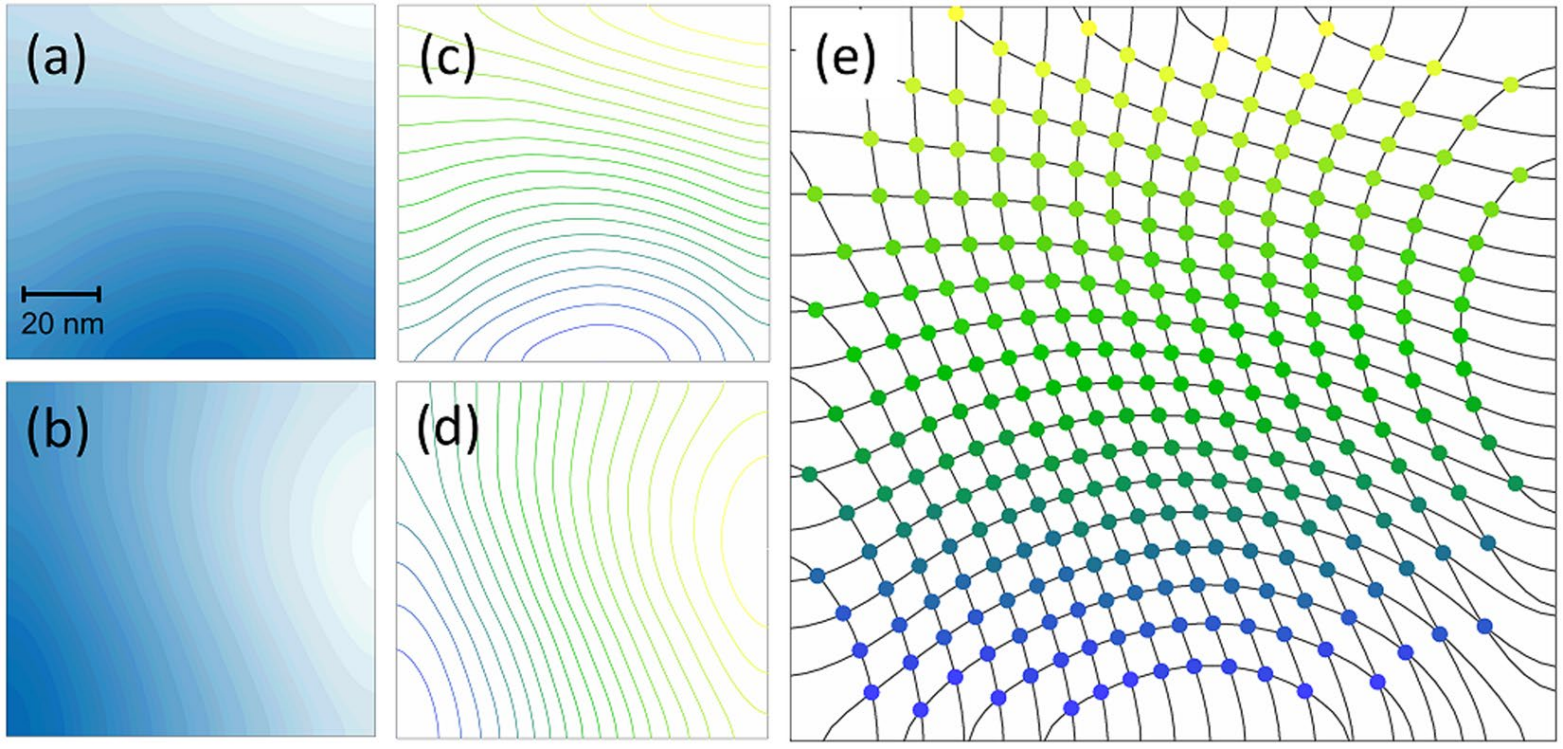
Tracking scheme used. A 200 nm × 200 nm scan, centered on the AFM tip apex is used for tracking tip motion. **(a)** Horizontal difference channel on the QAPD (left – right). **(b)** Vertical difference channel on the QAPD (bottom – top). The images are divided into evenly spaced contours (**c** and **d**), and the contour intersections are determined (**e**). These intersection positions are stored in FPGA memory in an order determined by the contour levels, so that real time QAPD signals can be readily converted to a memory index which holds the corresponding position.

Upon locating the AFM tip apex, we scanned a small area (100 nm × 100 nm) around this point with the piezo stage. The difference channels from the QAPD were digitized, normalized, and stored on the host computer for processing. The data was formatted to produce a 2D scan image, with the horizontal and vertical difference channels shown in Fig. 2(a) and (b) respectively. The full range of the scan was evenly divided into contour lines (Fig. 2(c) and (d)). The 2D position data associated with the intersections of these contour lines were stored on the FPGA to provide a fast conversion from normalized measurement to tip position (Supplementary Methods). The number of contours was optimized to provide high resolution within the constraint of FPGA memory utilization. For the results presented here, each 100 nm × 100 nm map was split into 200 contours, yielding sub-nanometer resolution (an average of 0.5 nm between contour intersections). Each contour’s position from the horizontal difference channel on the QAPD was checked against each vertical difference channel. Every intersection’s 16-bit X and Y coordinates were joined into a single 32-bit number and stored in order from top left to bottom right (Fig. 2(e)). Contours without an intersection used a placeholder value to maintain the order of the intersections. The end result of this process was an ordered 40,000 member (200 contours from each difference channel) 1D array, henceforth referred to as a “map”, which was stored on the FPGA’s memory for quick access.

For live tracking, the real-time normalized difference channel values were converted to an index in FPGA memory and the corresponding X, Y coordinates were retrieved from memory. This method minimizes the calculations required in the FPGA and maximizes the rate at which the position of the fiducial can be determined, producing a new position value at a rate of 1 MHz - limited by the digitization rate. In the FPGA logic we combine this with digitization-level averaging (100-point average of non-overlapping points) to reduce stochastic noise, which results in a position tracking rate of 8.6 kHz.

### Stabilizing fiducial position using feedback

The tracked fiducial position is used in the FPGA to calculate the deviation from a desired setpoint. Feedback commands to the sample stage are also generated on the FPGA and transmitted to the stage controller through a direct interface. By using an array of digital output lines from the FPGA, we control the position of the piezo sample stage, commanding movement at a rate up to the piezo controller’s servo update rate (20 kHz). This allows us to optionally maintain a desired position on the map by finding the displacement of the current tracked position from a desired position, and commanding the stage move to compensate for the measured displacement, forming a feedback loop. While commanding movement, we simultaneously poll the stage’s reported position. Completing these actions after a tracked position was calculated reduced the feedback rate to 5.9 kHz. The actual movement of the stage was slower due to the piezo’s physical stiffness limitation, with a resonant frequency of 340 Hz. Data on the tracked fiducial and stage position were periodically sent to the host computer through a set of buffers, decoupling the FPGA real-time processing from monitoring on the host computer.

### Active stabilization of single fluorescent molecules

To demonstrate our stabilization technique for fluorescent single molecules, we immobilized fluorescent streptavidin (Cy3-Sta) proteins on a glass coverslip. We first cleaned coverslips in a piranha solution (75% H_2_SO_4_: 25% H_2_O_2_) and glued an AFM chip in place using marine epoxy. We then placed a 100 µL drop of 1 g/L biotinylated bovine serum albumin (BSA-biotin) near the fiducial. The BSA readily adsorbs to the surface of the glass coverslip, while the attached biotin serves as a strong binding point for Cy3-Sta. We allowed the BSA-biotin to adsorb to the surface for 30 mins before rinsing the area with buffer. Next, we incubated the coverslip with 50 pM Cy3-Sta for 15 min. This protocol ensures optically resolvable binding of individual Cy3-Sta. After incubation we rinsed the coverslip twice and imaged in buffer by raster scanning the sample.

Because the feedback demonstrations took place over hours, we ensured the laser focus was maintained by locking the relative distance between the sample and the objective with the CRISP autofocus system (Applied Scientific Instrumentation, MS-2000 controller). This system uses an infrared LED inserted into the optical pathway to determine the distance between the objective and the sample, correcting any displacement by changing the voltage on a z-axis piezo (Physik Instrumente, PD72Z1CAQ) mounted between the microscope nosepiece and the objective.

## Results

We simultaneously obtained luminescence and backscatter images of the AFM tip (Fig. 1). The luminescence image of the AFM tip apex (Fig. 1(e)) was fit to a 2D Gaussian, which allowed us to accurately determine the apex position. Backscatter images of the pyramidal AFM tip, simultaneously generated by the QAPD and the APD (Fig. 1(c) and (d)), were similar and also permitted visualization of the tip apex. The tip apex determined from the backscattered images were within one standard deviation of a 2D Gaussian fit of the luminescence intensity, putting all three detection schemes in good agreement. For subsequent tracking and feedback, we used only the backscattered light from the fiducial.

**Figure 3:**
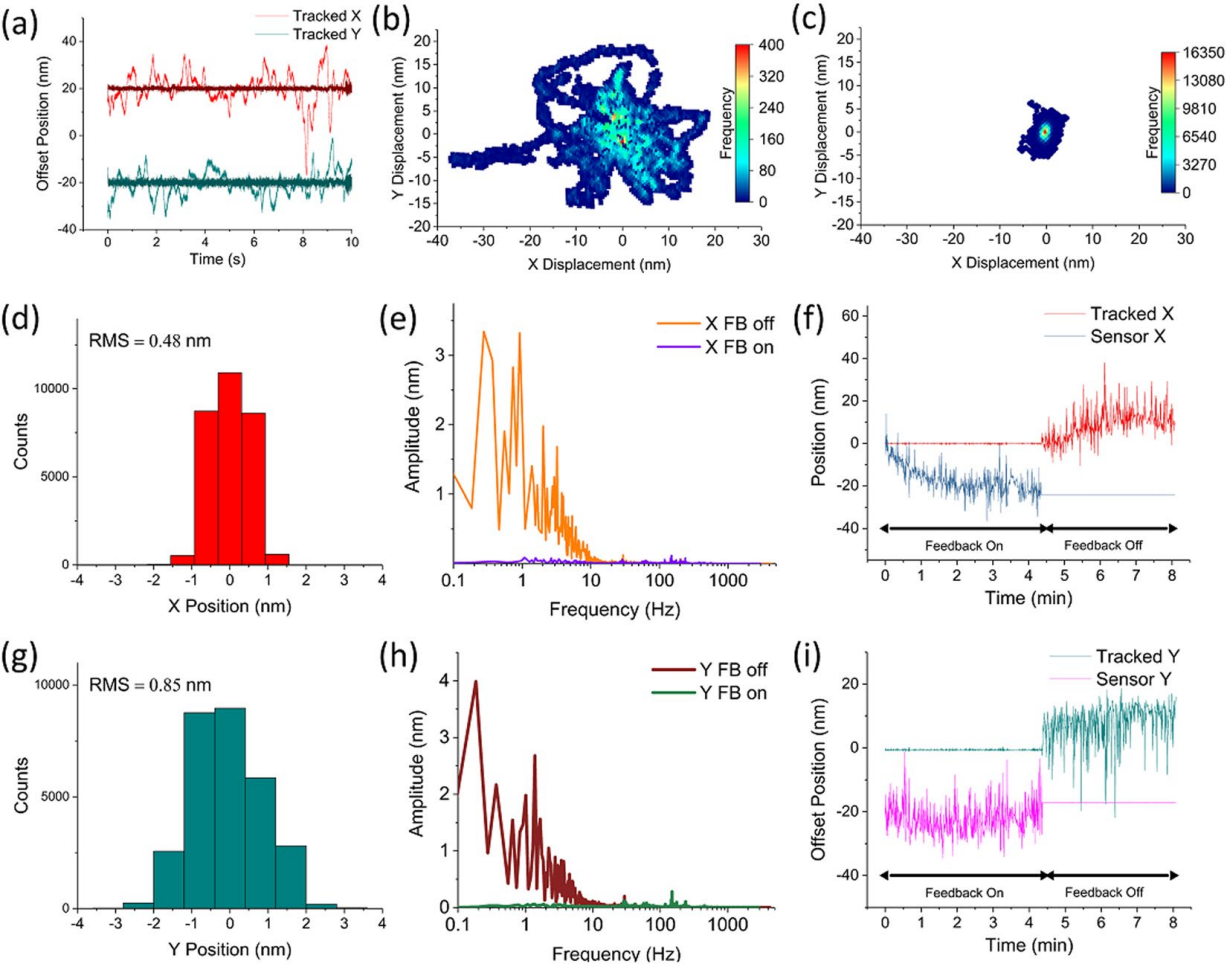
Demonstration of feedback. Picking a set-point position at the center of the scan, the tracking method is used to determine displacement from center and move the piezo stage to compensate for that displacement. **(a)** Position tracked for ten seconds in X (red) and Y (teal). Feedback is then enabled and resulting stabilized positions are shown overlaid and darker. Tracking with feedback off is collected at 8.6 kHz while tracking with feedback on is collected at 5.9 kHz. These tracked positions are shown in 2D in **(b)** with feedback off and **(c)** with feedback on. Projecting the tracked X data with feedback on yields the histogram shown in **(d)** with an RMS of 0.45 nm. This data is not fit to a Gaussian due to its discrete nature, as the tracked position will snap to the nearest grid point on the feedback map. **(e)** Fourier transforms of tracked X from (*a*), with feedback on and feedback off for ten seconds each. All large (>0.2 nm) amplitude fluctuations seen with feedback off are restricted to lower frequencies (under 20 Hz) and are eliminated with feedback on. **(f)** Tracked position was maintained for 4.5 minutes while feedback was turned on. The feedback was then switched off and the fiducial was allowed to drift for the remainder of the time (data averaged with a 100 point sliding window for clarity). The feedback compensates for long-term unidirectional drift of the sample, but also eliminates large short-term movement. Tracked position shown in red and piezo’s internal position sensor shown in blue. **(g)**, **(h)**, and **(i)** are the Y axis compliments of (*d*), (*e*), and (*f*). (**g**) Shows a wider spread in projected tracked values (0.85 nm), which can also be seen in (*c*). We attribute this difference to the non-linear nature of our feedback maps.

Using our tracking scheme, we tracked the 2D position of the tip apex in real-time at 8.6 kHz, and then turned on feedback and tracked the tip at 5.9 kHz (Fig. 3(a–c)). As Fig. 3(a) and (b) show, without feedback the tracked position varies as much as 55 nm in X and 35 nm in Y in only ten seconds. In contrast, with our feedback system active (Fig. 3(c)) the setpoint is maintained with sub-nanometer RMS along both axes, as shown in the projections (Fig. 3(d) and (g)). These histograms of the stabilized X and Y position monitored for ten seconds show a tight grouping around the zero setpoint (RMS = 0.48 nm and 0.85 nm, respectively). These values are consistent with the spacing between intersects during map generation and the fact that the relationship between tracked and stage coordinates is non-linear (see Fig. 2). Fourier transforms of the tracked data with feedback off and feedback on (Fig. 3(e) and (h)) demonstrate that fiducial motion with amplitudes exceeding ~0.2 nm (all in the lower frequency ranges) are effectively eliminated when feedback is enabled. To confirm our feedback method also corrects drift over longer time periods, we first tracked the AFM tip apex and used feedback to maintain the zero setpoint position for 4.5 minutes. We then turned feedback off and allowed the tip apex to drift for the remainder of the time (Fig. 3(f) and (i)). These data show a faithful handoff between stage movement and tracked position when feedback is toggled.

**Figure 4:**
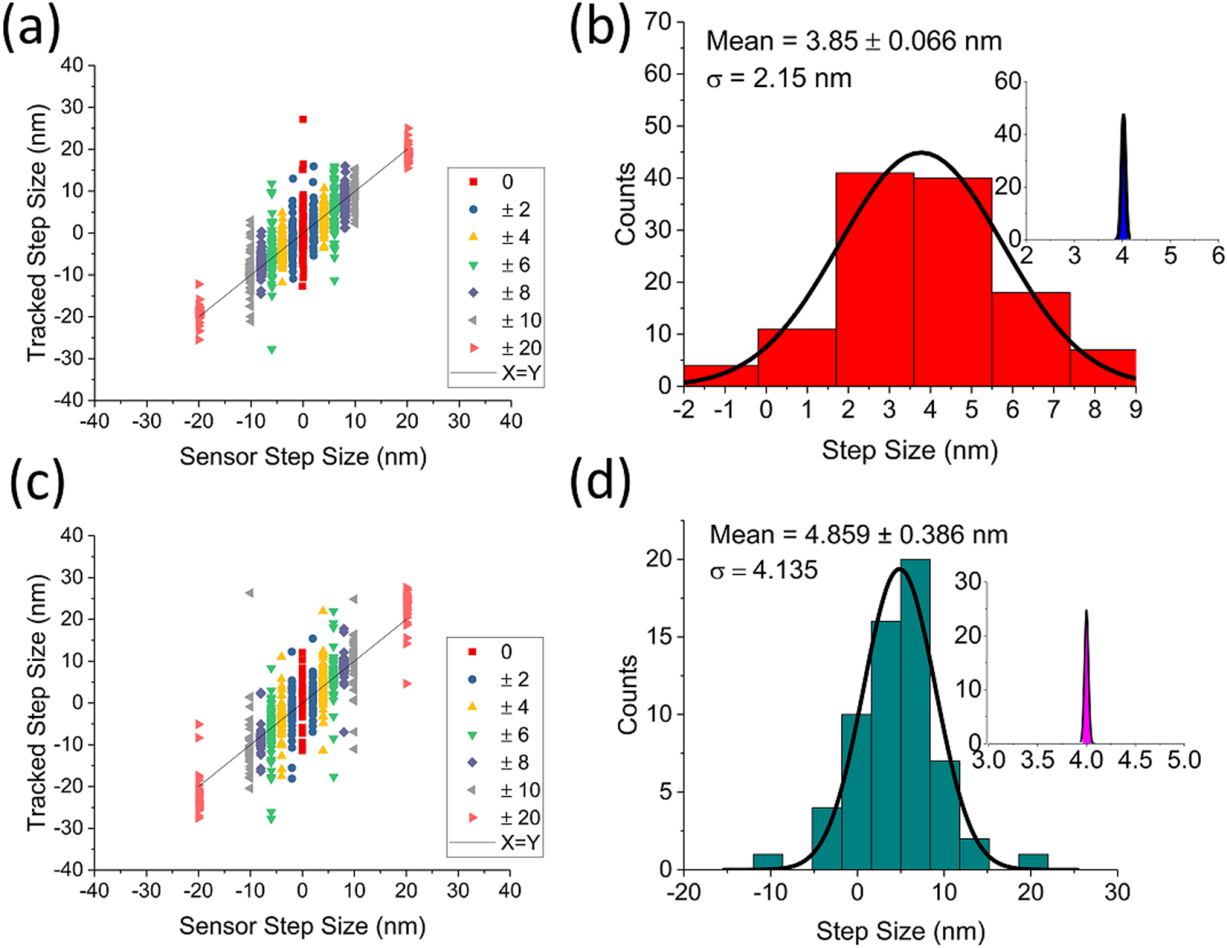
Correlating stage movement with tracked position. (**a**) The piezo stage was commanded to step forward and backward by a given distance along the X axis. At each position, 200 points were collected and averaged, effectively low-pass filtering the data to 43 Hz. The piezo’s internal position sensor (X axis) has a minimal spread corresponding to the stage’s specified accuracy, while tracked position is spread due to sample drift. **(b)** Representative step data (+4 nm step size) with corresponding piezo sensor data (inset). Sample drift over time moved the mean of the tracked step size 0.15 nm away from the commanded 4 nm. **(c)** Stepping was repeated along the Y axis. For these data, 700 points were collected and averaged at each position, effectively low-pass filtering the data to 12 Hz. **(d)** The +4 nm Y step size is projected, with drift contributing a 0.86 nm offset from the commanded 4 nm.

To correlate tracked position with stage location, we tracked the fiducial position while commanding the stage to step back and forth a set distance along each axis, with 17 ms wait between each position. First, we correlated tracked and stage sensor positions along the X axis. At each of 250 stage positions, 200 tracked and stage sensor positions were collected and averaged. The displacements between adjacent steps were recorded. This was done for 0 nm, 2 nm, 4 nm, 6 nm, 8 nm, 10 nm, and 20 nm step sizes, with the resulting displacements plotted in Fig. 4(a). As seen in the figure, the stage’s sensor displacement was tightly grouped for each step size while sample fluctuations caused the tracked displacement to vary a great deal. In this configuration, the mean of the tracked values is significantly shifted (0.15 nm) from the commanded 4 nm displacement due to the sample drift over the time required to collect the data (Fig. 4(b)). Next, we repeated the step tests for the Y axis while the X axis was held steady. For these tests, we collected and averaged 700 tracked and stage sensor positions for 125 movements with the same step distances (Fig. 4(c)). Along the Y axis the mean step-size is 0.86 nm greater than the commanded step-size of 4 nm (Fig. 4(d)).

**Figure 5:**
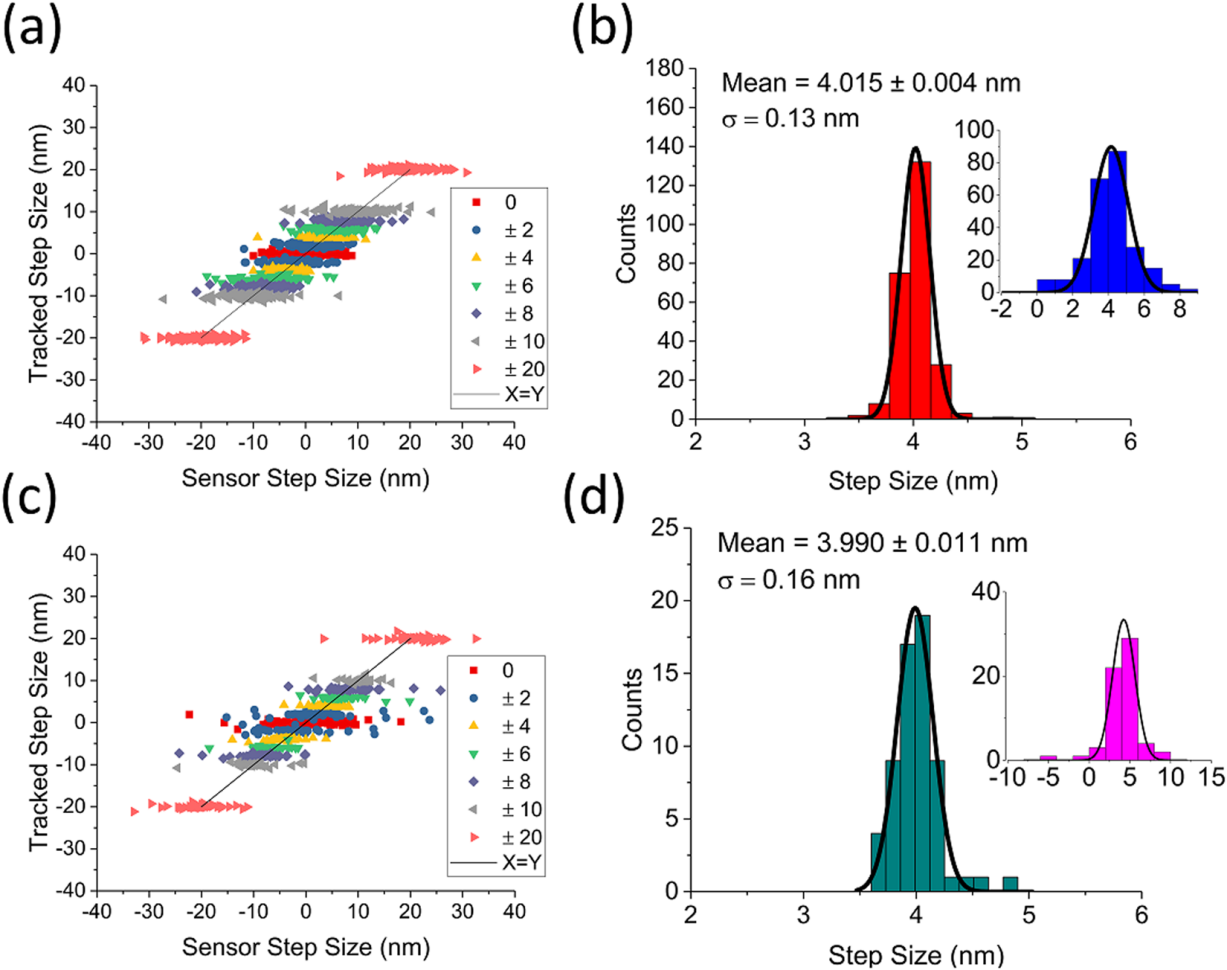
Benchmarking feedback accuracy and precision. (**a**) The step test shown in *4(a)* was repeated with feedback on. At each position, 200 points were collected and averaged, and with the lower acquisition rate the low pass cutoff was 29.5 Hz. **(b)** For direct comparison with *4(b)*, the projected and Gaussian fit +4 nm step data with corresponding piezo sensor data (inset) is shown. The spread in the tracked data is transferred to the sensor data as expected, and the difference between the requested step size and the actual tracked step size is only 15 ± 4 pm as the sample drift is continuously corrected, which sets the accuracy of our technique. The standard deviation of the measured step sizes is 130 pm, which sets our technique’s precision. **(c)** Stepping with feedback enabled was repeated along the Y axis. For these data, 700 points were collected and averaged at each position, effectively low-pass filtering the data to 8 Hz. **(d)** +4 nm Y step data with feedback on is projected and fit to a Gaussian, with a difference of only 10 ± 11 pm from the commanded 4 nm step size, and a standard deviation of 160 pm.

Next, to demonstrate our method’s accuracy and precision, the feedback was turned on and the experiment was repeated for the X axis with the feedback set-point varied instead of the commanded stage position. As expected, these results showed a tight grouping of tracked displacement while the sensor readout was spread as the stage moved to compensate for sample fluctuations (Fig. 5(a)). By projecting the +4 nm data from Fig. 5(a) and fitting the histogram to a Gaussian, we obtained a measure of the feedback accuracy and precision. With a commanded 4 nm movement, the tracked step size was peaked at 4.015 ± 0.004 nm, a difference of just 15 ± 4 pm which corresponds to feedback accuracy (Fig. 5(b)). The standard deviation of the measured step sizes was 130 pm, which demonstrates the precision of this technique relative to the sample stage. When these experiments were repeated along the Y axis (Fig. 5(c)), the corresponding accuracy and precision are 10 ± 11 pm and 160 pm, respectively (Fig. 5(d)).

**Figure 6:**
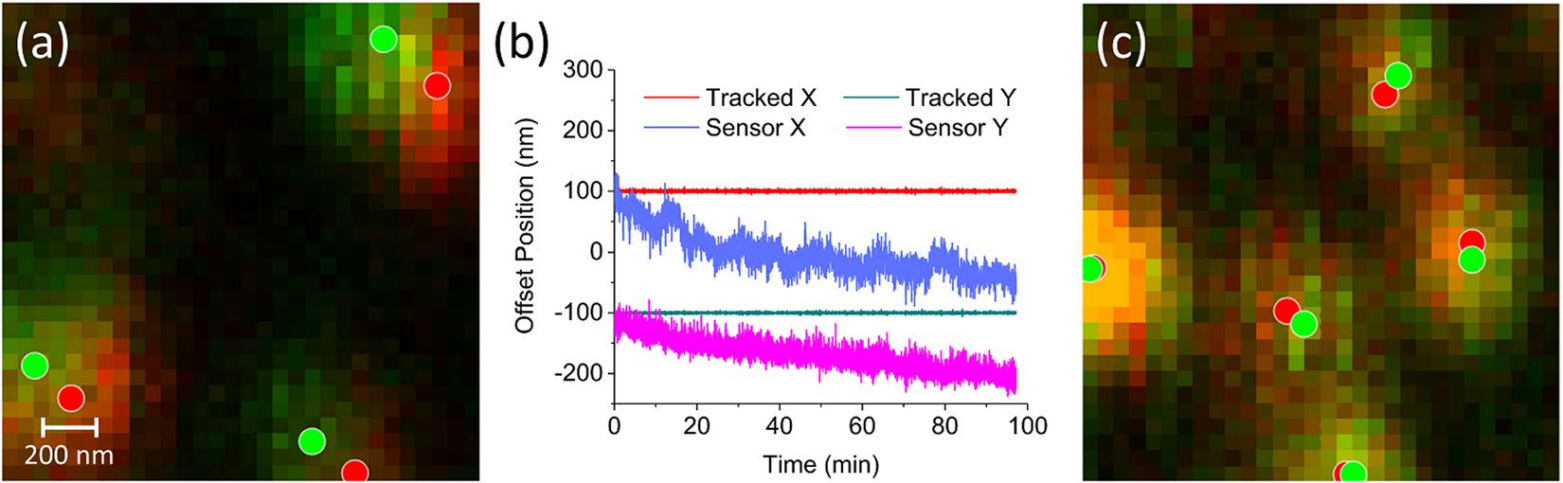
Active stabilization of single fluorescent molecules. Fluorescent Cy3-Sta was bound to the sample coverslip at a low enough density to allow resolution of individual molecules. **(a)** The same 1.8 µm × 1.8 µm region is scanned 100 minutes apart without feedback, and fluorescence is collected at APD 2. The initial scanned fluorescence image is shown in red, and the image scanned 100 minutes later is shown in green, with overlap shown in yellow. Each Cy3-Sta is fit to a 2D Gaussian for each scan and their centroids are plotted as red and green circles. The average displacement of these molecules (average of eight centroids from a wider view) after drift was 4.9 pixels in X and 3.02 pixels in Y. This process was repeated for a new region with feedback enabled for 100 minutes, resulting in the tracked and stage sensor values plotted in **(b**) and the initial and final images with centroids shown in **(c)**. With feedback enabled the mean of the centroid differences (average of eleven such centroids from a wider view) is reduced to 0.4 pixels in X and 0.06 pixels in Y.

With the feedback method verified and benchmarked, we sought to stabilize single fluorescent biomolecules under physiologically relevant conditions. As described in the methods section, we immobilized optically well separated Cy3-Sta on our sample. We imaged fluorescent molecules near our fiducial and then allowed the system to drift for 100 minutes. We scanned the area a second time, and the resulting Cy3-Sta displacement can be seen in the overlaid image, with markers on the fluorophores’ centroid determined by a 2D Gaussian fit (Fig. 6(a)). By taking the difference of eight such centroids from a wider view, before and after drift, we determined the drift is 4.9 pixels in X and 3.02 pixels in Y (pixel size = 60 nm × 60 nm). Next, we imaged a new set of Cy3-Sta near the fiducial, after which feedback was enabled for 100 minutes before rescanning the area. The stage sensor values in Fig. 6(b) show the 2D drift correction during this time. The second set of overlaid images with fitted centroids are shown in Fig. 6(c). The average of eleven centroid displacements was only 0.4 pixels in X and 0.06 pixels in Y. It is important to note that since feedback was turned off during image acquisition, centroids measured in subsequent images were offset due to sample drift. During the 3 seconds it takes to image a single Cy3-Sta, a sample can drift up to 55 nm (Fig. 3(a) and (b)) which may account for the tiny centroid displacements measured in the presence of feedback (Fig. 6(c)).

## Discussion

The data presented here demonstrate a feedback method that maintains fiducial location with sub-nanometer accuracy and precision while using low intensity laser light. This configuration is ideal for single molecule fluorescence measurements. Because our resolution is currently limited by the resolution of the map, the effective resolution of our technique can be easily increased by either increasing the number of contours used to find intersections or decreasing the map scan area. Ultimately, the FPGA onboard memory limits how many intersections can be stored, but an optimal map can be determined by balancing desired travel range with intersect density.

To implement our method in AFM applications, feedback in three dimensions is necessary. It is important to point out that our method is not fundamentally limited to two dimensions. We can extend our method to stabilize a sample in 3D, for instance, by utilizing a set of maps at different heights along the optical axis.

While we tracked the position of the fiducial at a rate of 8.6 kHz, our tracking speed was limited by the fact that we used an average of 100 consecutive digitized points to reduce noise in the tracked position. At the possible expense of a lowered signal to noise ratio, reducing the number of averages or eliminating them altogether could greatly increase the tracking speed. The amount of averaging can be tuned to optimize the method for different applications.

We anticipate that the feedback technology presented here has broad applications in stabilizing instruments that combine single molecule AFM and fluorescence-based approaches. An existing version of this instrument, termed ‘single-molecule cut-and-paste’^12^, has been used to construct biological nanostructures by using a specially functionalized AFM tip to move molecules from a reservoir to a target area, one molecule at a time. Our feedback method will allow the AFM tip to repeatedly address the same location several times, potentially constructing more complex structures. By addressing specific locations or fiducials, the placement of molecules would also be specific, eliminating the need to ‘blindly’ pick up and place molecules over a large area.

Our method also has broad applications in stabilizing conventional fluorescence microscopes. In these microscopes, long term molecular dynamics are visualized by taking repeated fluorescence images, while utilizing feedback methods to stabilize the microscope so that the molecules do not drift out of view. Unlike other ultrastable platforms which employ a high laser intensity that would photobleach the sample, our method uses lower light intensity. We have demonstrated our method can stabilize individual fluorescent molecules without photobleaching them.

In addition to stabilizing microscope platforms, our method has broad applications in tracking the motion of single molecules *in vitro* and in live-cells. As many biological processes rely on sub-millisecond motions of single molecules, the high tracking rate presented here would allow these movements to be monitored with high temporal resolution. For instance, protein movement has previously been tracked by covalently attaching gold nanoparticles (GNPs) to a protein of interest and monitoring backscattered light on a QPD^27^. While this method boasts an impressive 40 kHz tracking rate with 1.5 nanometer resolution, the tracking is not conducted in “real-time” since the position data cannot be retrieved until the experiment is completed. In contrast, our tracking method allows for sub-nanometer tracking resolution and real-time position information. Furthermore, our low laser intensity operation would allow this sort of tracking to be done in conjunction with fluorescence microscopy techniques.

## Electronic supplementary material

Supplementary Information
