# Supplementary material for: Method for high frequency tracking and sub-nm sample stabilization in single molecule fluorescence microscopy
Source: Sci Rep. 2018 Sep 17;8:13912. doi: 10.1038/s41598-018-32012-1 (PMC6141618; doi:10.1038/s41598-018-32012-1)
Supplement: Supplementary file 1 — Supplementary Information [file 41598_2018_32012_MOESM1_ESM.pdf]

Supplemental Information

Title: Method for high frequency tracking and sub-nm sample stabilization in single molecule fluorescence microscopy

Authors: Patrick D. Schmidt [1], Benjamin H. Reichert [1], John G. Lajoie [2], and Sanjeevi Sivasankar [1, 2, 3, *]

Affiliation: [1] Department of Electrical and Computer Engineering; [2] Department of Physics and Astronomy, Iowa State University, Ames, IA 50011, USA.

Current Affiliation: [3] Department of Biomedical Engineering, University of California, Davis, CA 95616, USA.

***Correspondence to ssivasankar@ucdavis.edu**

Supplementary Methods

Tracking scheme: For tracking we need to use the real time QAPD values to find the corresponding position values stored in the FPGA memory. We convert real-time QAPD values via the equation:

$$Index\ = (LR - LRmin\ ) \times LRslope + (BT - BTmin) \times BTslope \times n$$

where *LR* and *BT* are the real time difference channel values, *LRmin* and *BTmin* are the lowest values from the difference channel maps, *n* is the number of contours per channel, and *LRslope* is calculated by:

$$LRslope = \frac{n - 1}{LRmax - LRmin}$$

where *LRmax* is the highest value from the horizontal difference channel map. *BTslope* is calculated similarly to *LRslope*. This index holds a 32 bit number which is split to obtain the 16 bit X and Y position.
